# DNA methylation profiling identifies two distinct subgroups in breast cancers with low hormone receptor expression, mainly associated with *HER2* amplification status

**DOI:** 10.1186/s13148-021-01176-5

**Published:** 2021-10-03

**Authors:** Philipp Jurmeister, Karsten Weber, Sonia Villegas, Thomas Karn, Michael Untch, Anne Thieme, Volkmar Müller, Eliane Taube, Peter Fasching, Wolfgang D. Schmitt, Frederik Marmé, Elmar Stickeler, Bruno V. Sinn, Paul Jank, Christian Schem, Frederick Klauschen, Marion van Mackelenbergh, Carsten Denkert, Sibylle Loibl, David Capper

**Affiliations:** 1 Institute of Pathology, Charité - Universitätsmedizin Berlin, Freie Universität Berlin, Humboldt-Universität zu Berlin and Berlin Institute of Health, 10117 Berlin, Germany; 2 German Cancer Consortium (DKTK), Partner Site Berlin, German Cancer Research Center (DKFZ), 69210 Heidelberg, Germany; 3 Berlin Institute of Health, Anna-Louisa-Karsch-Straße 2, 10178 Berlin, Germany; 4 German Breast Group, 63263 Neu-Isenburg, Germany; 5 Department of Gynecology and Obstetrics, Goethe University, Frankfurt, Germany; 6 Department of Gynecology and Obstetrics, Breast Cancer Center, Helios-Klinikum Berlin, Buch, Germany; 7 Department of Obstetrics and Gynecology, Universitätsklinikum Hamburg-Eppendorf, Hamburg, Hamburg, Germany; 8 Brustzentrum, Universitätsklinikum Erlangen, Erlangen, Germany; 9 Department of Obstetrics and Gynecology, University Hospital Heidelberg, Heidelberg, Germany; 10 Klinik für Gynäkologie und Geburtsmedizin, Universitätsklinikum Aachen, Aachen, Germany; 11 Institute of Pathology, Philipps-University Marburg and University Hospital Marburg, Marburg, Germany; 12 Mammazentrum Hamburg, Hamburg, Germany; 13 University Hospital of Schleswig-Holstein, Campus Kiel, Germany; 14 Department of Neuropathology, Charité - Universitätsmedizin Berlin, Freie Universität Berlin, Humboldt-Universität zu Berlin, 10117 Berlin, Germany; 15 Institute of Pathology, Ludwig Maximilians University Hospital Munich, Thalkirchner Str. 36, 80337 Munich, Germany

## Abstract

**Background:**

Current clinical guidelines suggest that breast cancers with low hormone receptor expression (LowHR) in 1–10% of tumor cells should be regarded as hormone receptor positive. However, clinical data show that these patients have worse outcome compared to patients with hormone receptor expression above 10%. We performed DNA methylation profiling on 23 LowHR breast cancer specimens, including 13 samples with *HER2* amplification and compared our results with a reference breast cancer cohort from The Cancer Genome Atlas to clarify the status for this infrequent but important patient subgroup.

**Results:**

In unsupervised clustering and dimensionality reduction, breast cancers with low hormone receptor expression that lacked *HER2* amplification usually clustered with triple negative breast cancer (TNBC) reference samples (8/10; “LowHR TNBC-like”). In contrast, most specimens with low hormone receptor expression and *HER2* amplification grouped with hormone receptor positive cancers (11/13; “LowHR HRpos-like”). We observed highly similar DNA methylation patterns of LowHR TNBC-like samples and true TNBCs. Furthermore, the Ki67 proliferation index of LowHR TNBC-like samples and clinical outcome parameters were more similar to TNBCs and differed from LowHR HRpos-like cases.

**Conclusions:**

We here demonstrate that LowHR breast cancer comprises two epigenetically distinct groups. Our data strongly suggest that LowHR TNBC-like samples are molecularly, histologically and clinically closely related to TNBC, while LowHR HRpos-like specimens are closely related to hormone receptor positive tumors.

**Supplementary Information:**

The online version contains supplementary material available at 10.1186/s13148-021-01176-5.

## Background

The classification of breast cancer is based on the expression of the estrogen and progesterone receptor as well as the presence or absence of *HER2* amplification [1]. Patients with hormone receptor positive tumors usually benefit from endocrine therapy and have better disease specific survival compared to hormone receptor negative tumors [2]. Additionally, tumors with *HER2* amplification can be treated with different types of anti-*HER2*-therapy [3].

The evaluation of hormone receptor expression and the *HER2* status is primarily performed by using immunohistochemistry (IHC) and in situ hybridization. In the 2010 revision of the guidelines of the American Society of Clinical Oncology and the College of American Pathologists (ASCO/CAP), the cut-off value for the definition of hormone receptor positive breast cancers was reduced from 10 to 1% [4]. Only recently, the ASCO/CAP has updated this assessment, but also introduced a “ER low positive” category for samples with estrogen receptor expression between 1 and 10% [5]. However, breast cancers with low hormone receptor expression are usually still considered as hormone receptor positive (= luminal) subtype [6]. Various studies showed that therapy response and outcome of patients with tumors showing low hormone receptor expression between 1 and 10% (LowHR) are more similar to triple negative breast cancer (TNBC), the so-called basal subtype [6–13]. These results are supported by molecular studies, using RNA-based classification algorithms [14]. This led to a dilemma in treatment decision, as it is unclear if patients with LowHR tumors should receive endocrine therapy or not or should be treated with more aggressive approaches like adjuvant chemotherapy, including platin compounds and even more, whether these tumors might be candidates for targeted therapeutic approaches like CDK4/6 inhibitors. More importantly, these patients are typically not included in clinical trials investigating new therapy strategies for TNBC, like immune checkpoint inhibitors. Furthermore, there are no data on the treatment of low hormone receptor expression and the additional *HER2* amplification.

More recently, numerous studies showed that DNA methylation profiles are a reliable tool to classify different cancer types, reflecting the tumor’s cell-of-origin [15–17]. With regards to breast cancer, TNBCs exhibit global hypomethylation which is distinct from hormone receptor positive tumors [18]. To find new molecular support for the currently proposed hypothesis that breast cancers with low hormone receptor expression resemble TNBCs, we subjected a cohort of these two subgroups to genome-wide DNA methylation profiling and compared the epigenetic profile of these specimens with a large reference cohort of hormone receptor positive tumors and TNBCs from The Cancer Genome Atlas (TCGA). Furthermore, we also correlated our results with clinical patient outcome.

## Results

### Dimensionality reduction and cluster analysis

**Fig. 1:**
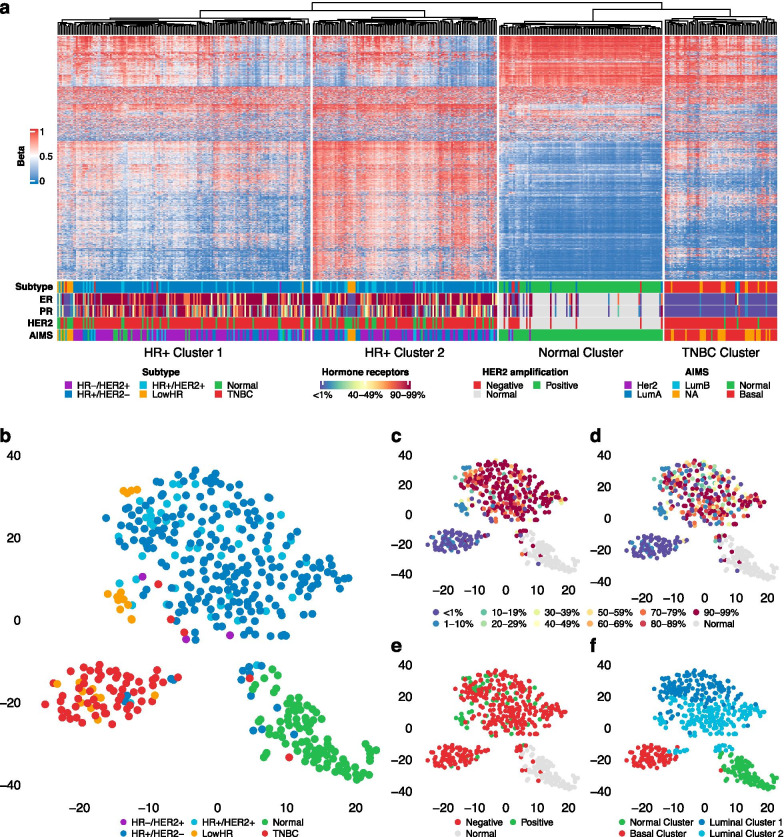
Unsupervised hierarchical clustering as well as t-distributed stochastic neighbor embedding (t-SNE) to compare DNA methylation signatures across different breast cancer subtypes from The Cancer Genome Cohort (*n* = 422) as well as our own analyses (*n* = 36). **a** Unsupervised hierarchical clustering reveals four distinct clusters, representing normal breast tissue (“Normal Cluster”), tumors with hormone receptor expression (“HR+ Cluster 1” and “HR+ Cluster 2”) as well as TNBCs (“TNBC Cluster”). Molecular subtype classification based on results from immunohistochemistry (IHC) and/or HER2 in situ hybridization as well as RNA sequencing using the AIMS classification are shown below the heatmap. **b** General annotation of normal breast tissue and different breast cancer subtypes from IHC and/or in situ hybridization in a t-SNE plot. **c** and **d** t-SNE plot showing the distribution of estrogen (**c**) and progesterone (**d**) receptor expression. **e** t-SNE visualizing the *HER2* amplification status. **f** Correlation between t-SNE analysis and hierarchical clustering. Abbreviations: *ER* estrogen receptor, *PR* progesterone receptor

Unsupervised hierarchical clustering of LowHR and TCGA samples based on DNA methylation profiles revealed four distinct clusters (Fig. 1a), mainly representing normal breast tissue (“Normal Cluster”), hormone receptor positive breast cancers (“HR+ Cluster 1” and “HR+ Cluster 2”) as well as TNBCs (“TNBC Cluster”) with little overlap between the different groups. The two HR+ clusters were not associated with the luminal A or B subgroups as assessed using the RNA sequencing-based AIMS classification scheme. Similar results were seen using a t-SNE analysis (Fig. 1b–f), although there was no clear separation between the two groups of hormone receptor positive tumors that were observed in hierarchical clustering (Fig. 1f). Tumors in the periphery of their respective groups as well as tumor samples that fell in the normal breast tissue group tended to have relatively low tumor cell content (Additional file 1: Fig. S1).

Specimens with low hormone receptor expression either fell into one of the HR+ clusters or the TNBC cluster. This separation was mainly associated with the *HER2* amplification status: samples that clustered with hormone receptor positive tumors (LowHR HRpos-like) usually showed *HER2* amplification (11/13; 85%), while this was a rare event in LowHR TNBC-like samples (2/10; 20%). Of note, the *HER2* status in copy number data derived from DNA methylation data was in line with the results from IHC and/or SISH. In particular, we confirmed the presence of *HER2* amplification in the two *HER2* positive LowHR TNBC-like samples and did not find any evidence for *HER2* copy number changes in the two *HER2* negative LowHR HRpos-like samples.

**Fig. 2:**
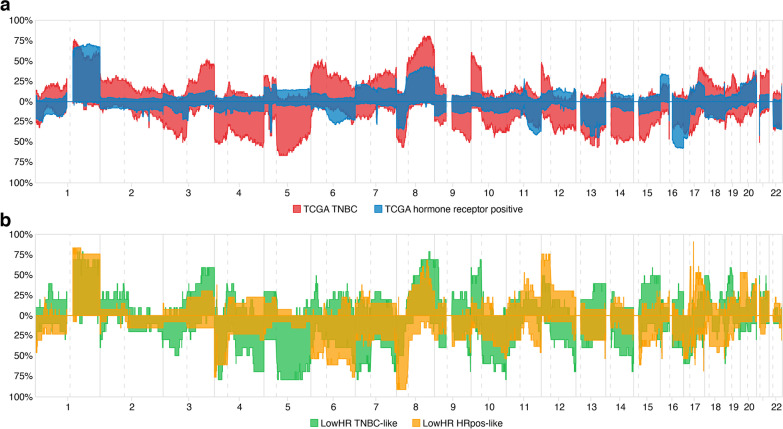
Summary copy number plots derived from DNA methylation data, showing the proportion of tumor samples with gains (above the baseline) or losses (below the baseline) at the respective position. **a** Summary copy number plot of hormone receptor positive (*n* = 282) and triple negative samples (TNBC; *n* = 52) from The Cancer Genome Atlas (TCGA). **b** Summary copy number plot comparing genome wide copy number profiles of samples with low hormone receptor expression that clustered with hormone receptor positive (LowHR HRpos-like; *n* = 13) or triple negative breast cancers (LowHR TNBC-like; *n* = 10) in unsupervised hierarchical cluster analysis. The focal spike at chromosome 17q in the LowHR HRpos-like subgroup represents the gene locus of *ERBB2*

Summary copy number plots derived from DNA methylation data revealed additional similarities between LowHR TNBC-like samples and TNBCs, such as frequent losses of chromosome 4q and 5q as well as recurrent gains of chromosome 10p (Fig. 2).

### Analysis of differential DNA methylation

To compare DNA methylation profiles between TNBCs and the two LowHR subtypes, we tested our dataset for differentially methylated positions (DMP) and differentially methylated regions (DMR).

**Fig. 3:**
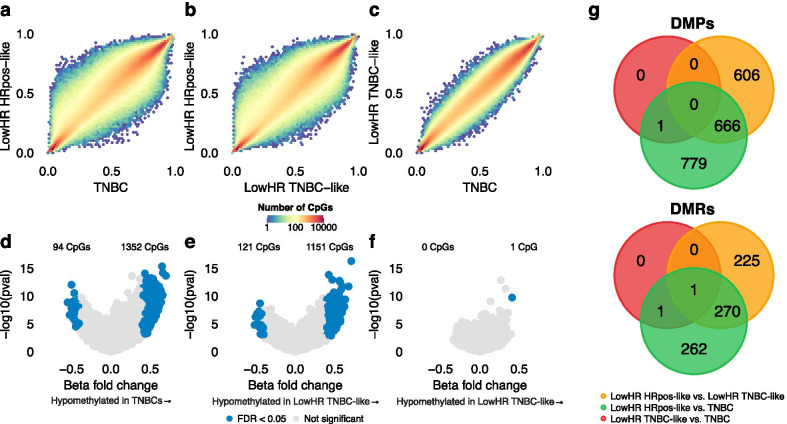
Density plots, volcano plots and Venn diagrams to compare beta values, differentially methylated positions (DMP) and differentially methylated regions (DMR) between triple negative breast cancers (*n* = 14) and the two subtypes with low hormone receptor expression (LowHR; *n* = 23). **a**–**c** Density plots showing the pairwise correlation of beta values of all CpGs between the three subgroups. **d**–**f** Volcano plots visualizing DMPs between LowHR HRpos-like and TNBC samples (**d**), LowHR HRpos-like and LowHR TNBC-like specimens (**e**) as well as TNBC and LowHR TNBC-like samples (**f**). **f** Venn diagrams showing overlapping and unique DMPs and DMRs between the pairwise comparisons

A correlation analysis of the beta values of all CpGs revealed general hypermethylation in LowHR HRpos-like samples when compared to LowHR TNBC-like and TNBC specimens (Fig. 3a, b). There was a very strong, almost linear correlation between LowHR TNBC-like and TNBC samples (Fig. 3c). We identified 1446 DMPs between TNBC and LowHR HRpos-like samples. 1352 CpGs (93%) were hypomethylated in TNBCs (Fig. 3d). Comparing LowHR TNBC-like with LowHR HRpos-like samples, 1272 site were significantly differentially methylated. Again, the majority of CpGs (1151, 89%) were hypomethylated in LowHR TNBC-like samples (Fig. 3e). There was a considerable overlap between the identified DMPs, with 666 shared CpGs sites (Fig. 3g). Furthermore, we observed almost no difference between LowHR TNBC-like and TNBC samples, as our analysis only revealed a single differentially methylated probe (Fig. 3f). Similar results were obtained for DMRs. The DMRs identified when comparing LowHR HRpos-like samples and TNBCs included cancer-relevant genes such as *EN1*, *TFF3* or *IRX1* which have previously been described to be differentially methylated and expressed in TNBCs and hormone receptor positive breast cancers [26–28]. All DMRs are listed in Additional files 5–7: Tables S3–S5.

### Correlation with proliferation index and clinical outcome

To further evaluate potential differences regarding the biological behavior and clinical outcome of the two identified LowHR subtypes and TNBCs, we compared the Ki67 tumor proliferation rate as well as pCR rate and OS between these groups using clinical trial information. DNA methylation classes did not correlate with local tumor stage or nodal status (Additional file 8: Table S6).

**Fig. 4:**
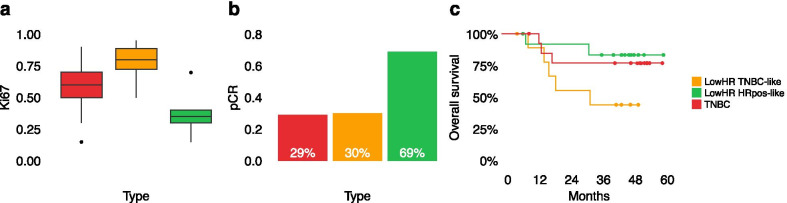
Correlation of the two LowHR subtypes (*n* = 23) and triple negative breast cancers (TNBC; *n* = 14) with proliferation rate and clinical outcome. **a** Boxplot comparing the Ki67 proliferation rate between the three groups. **b** Barplot comparing the pathological complete response of the two LowHR subgroups and TNBCs. **c** Kaplan–Meier plot showing overall survival rates across the three groups

With 56% (*p* = 0.03) in TNBCs and 77% (*p* = 0.0002) in LowHR TNBC-like samples, the proliferation rate (Ki67) of these groups was significantly higher than in LowHR HRpos-like tumors (37%; Fig. 4a).

Furthermore, the pCR rate in TNBC (4 of 14 patients, 29%) and LowHR TNBC-like samples (3 of 10 patients, 30%) was lower than in LowHR HRpos-like cases (9 of 13 patients, 69%), although this difference had only borderline significance (*p* = 0.05 and *p* = 0.09; Fig. 4b). Notably, OS tended to be shorter in patients with LowHR TNBC-like tumors, although pairwise log rank test was not significant (*p* = 0.07) when compared with LowHR HRpos-like samples (Fig. 4c).

## Discussion

With this study, we demonstrate that breast cancers with low hormone receptor expression can be separated into two highly distinct groups by DNA methylation profiling. One group (“LowHR HRpos-like”) shows a DNA methylation profile similar to hormone receptor positive tumors, is strongly enriched with *HER2* amplification, closely mirrors the chromosomal pattern of hormone receptor positive tumors and has a lower proliferation rate and a better clinical course. The other group (LowHR TNBC-like) shows a DNA methylation pattern of TNBC, has low rates of *HER2* amplification, closely resembles the chromosomal pattern of TNBC and has a high proliferation rate and worse clinical outcome.

Since the lowering of the cut-off value for hormone receptor positive breast cancers by the ASCO/CAP in 2010, repeated concerns were raised that the outcome of patients with LowHR tumors is worse, as they do not benefit from endocrine therapy. However, despite from RNA expression profiles, there were no molecular data to support this clinical observation [14].

Using hierarchical clustering as well as t-SNE as a method of dimensionality reduction that has previously been proven to be a valuable tool to identify subgroups in DNA methylation data sets, we observed that normal breast tissue, hormone receptor positive breast cancers and TNBCs can be reliably distinguished based on their epigenetic profile. The small overlap between tumor samples and normal breast tissue was most likely due to low tumor cell content within these specimens, as we have recently demonstrated for other tumor entities [17]. The overlap between the two hormone receptor positive and the TNBC cluster was also very low. This is in line with previous studies that demonstrated that TNBCs exhibit epigenetic signatures characterized by global DNA hypomethylation that are distinct from hormone receptor positive breast cancers [18]. These findings provided the necessary rationale to compare the epigenetic signatures of these two subtypes with LowHR specimens. As described, LowHR tumors lacking *HER2* amplification predominantly clustered with TNBCs, while specimens with additional *HER2* amplification were typically very similar to hormone receptor positive tumors. Four specimens did not fit into this pattern. Differential DNA methylation analysis revealed almost no difference between LowHR TNBC-like and true TNBC samples, strongly supporting the assumption that these subtypes do not only show similar clinical outcome, but are also identical on the molecular level. Our investigation for differentially methylated genes between LowHR HRpos-like and TNBC specimens revealed a considerable overlap with cancer-relevant genes that have previously been described to be differentially methylated and expressed in hormone receptor positive tumors and TNBCs [26]. This indicates that despite the low expression of hormone receptors, most *HER2* amplified tumors are still very similar to hormone receptor positive breast cancers.

As DNA methylation signatures are considered to represent the cell-of-origin of a tumor, these findings could indicate that LowHR TNBC-like samples derive from the same cell type as TNBCs. On the other hand, the presence of *HER2* amplification in LowHR HRpos-like specimens suggests a common origin with hormone receptor positive breast cancer, in line with previous cell-of-origin theories [29]. This is also supported by the observation that all three hormone receptor negative samples with *HER2* amplification that were included in the TCGA reference data set aggregated in one of the HR+ clusters.

Using genome wide copy number profiles derived from raw DNA methylation data, we were also able to study the differences in frequency of genome copy number alterations between hormone receptor positive tumors, TNBCs and LowHR specimens. In line with our results from DNA methylation profiling, LowHR TNBC-like tumors and TNBCs generally showed comparable numbers of recurrent genome copy abnormalities. On the other hand, copy number profiles of LowHR HRpos-like samples generally resembled hormone receptor positive breast cancers, with the exception of the previously described high rate of *HER2* amplification. Previous studies already showed that there are differences in frequency of numeric alterations between TNBCs and hormone receptor positive tumors and the respective chromosomal locations we observed in our study were in line with these reports [30, 31]. These findings provide additional and independent proof for the correctness of the DNA methylation-based classification of LowHR tumors.

Based on our results, DNA methylation-based classification of LowHR tumors could be used to assess the individual prognosis and could guide treatment decisions in further studies. Based on the differences in their epigenetic profiles and clinical outcome data, one might suggest that LowHR TNBC-like samples require a similar treatment as TNBCs, whereas patients with LowHR HRpos-like tumors might still benefit from endocrine therapy. Strikingly, we observed high pCR rates for LowHR HRpos-like samples, which is unexpected due to their molecular similarity with hormone receptor positive breast cancer. However, it remains uncertain if this behavior is characteristic for this specific subgroup or if this observation is biased by other factors. Due to the retrospective design of our study and the small sample size, the clinical results should be interpreted with caution and need to be addressed in larger and prospective clinical trials.

## Conclusion

In conclusion, using DNA methylation our study provides new molecular support that LowHR breast cancer comprises two molecularly distinct groups that can be separated by DNA methylation profiling, resembling TNBC and hormone receptor positive breast cancer with potential implications for individual patient prognosis and therapy selection.

## Methods

### Patients and samples

A flowchart which summarizes the composition of the study cohort is available as Additional file 2: Fig. S2.

For this study, we selected a total of 23 cases from the GeparSixto (NCT01426880) and GeparSepto (NCT01583426) trial of the German Breast Group (GBG) and the Arbeitsgemeinschaft Gynäkologische Onkologie – Breast Study Group (AGO-B) with estrogen and progesterone receptor expression between 1 and 10%, including 13 samples with HER2 amplification [19, 20]. Informed consent was obtained from all patients for study participation and translational research projects. Histopathological characteristics are summarized in Table 1. As a reference, we also included 14 randomly selected TNBC specimens from the GeparSepto study with estrogen and progesterone receptor expression < 1%. This study included only female early stage breast cancer patients without distant metastases. For all analyses, formalin-fixed and paraffin-embedded biopsy specimens from the initial diagnosis were used. Therefore, all patients were treatment-naïve. All samples were tested for hormone receptor expression and *HER2* status as part of the central pathology assessment at the Institute of Pathology of the Charité – University Hospital Berlin.

**Table 1:** Table summarizing the proportion of estrogen (ER) and progesterone receptor (PR) positive tumor cells, the results from HER2 immunohistochemistry (IHC) and silver in situ hybridization (SISH), the Ki67 proliferation rate as well as histopathological grading of LowHR samples included in this study

Case #	ER (%)	PR (%)	HER2 IHC	HER2 SISH	Ki67 (%)	Grading	cT	cN
1	8	0	1+	Not done	80	G3	cT2	cN−
2	5	0	3+	Not done	30	G3	cT2	cN+
3	2	0	3+	Positive	15	G2	cT2	cN+
4	5	0	2+	Positive	40	G3	cT2	cN+
5	5	0	3+	Positive	15	G2	cT2	cN−
6	5	0	3+	Positive	40	G3	cT2	cN−
7	5	0	3+	Not done	30	G3	cT2	cN+
8	0	3	1+	Not done	90	G3	cT1	cN−
9	5	0	2+	Negative	70	G3	cT2	cN−
10	0	5	3+	Not done	70	G3	cT2	cN−
11	3	0	0	Not done	80	G3	cT3	cN+
12	5	0	0	Not done	80	G3	cT1	cN+
13	< 1	8	3+	Not done	20	G3	cT4	cN+
14	5	0	0	Not done	50	G3	cT1	cN+
15	8	8	3+	Not done	73	G3	cT2	cN+
16	2	0	2+	Negative	50	G3	cT2	cN−
17	3	2	2+	Negative	85	G3	cT1	cN−
18	3	< 1	3+	Not done	40	G3	cT2	cN−
19	3	0	2+	Negative	30	G3	cT2	cN−
20	0	5	0	Not done	95	G3	cT2	cN−
21	2	0	2+	Positive	90	G3	cT2	cN−
22	0	5	3+	Not done	38	G3	cT2	cN−
23	2	0	3+	Not done	35	G3	cT2	cN+

Raw DNA methylation data from 709 samples as well as the corresponding pathology reports were obtained from the TCGA BRCA dataset available at the TCGA legacy archive (https://portal.gdc.cancer.gov/legacy-archive/). In this study, the definition of different breast cancer subtypes is based on the results from IHC. Therefore, we excluded patient samples without documented percentage of estrogen or progesterone receptor positive cells by IHC (*n* = 271) in the corresponding pathology reports as well specimens with bad quality in DNA methylation analysis (*n* = 16), as defined below. The resulting reference dataset consisted of 422 samples, including 334 breast cancer and 88 normal breast tissue specimens. The corresponding sample list is available as Additional file 3: Table S1.

### IHC and silver in situ hybridization (SISH)

Antibodies, SISH reagents and scoring systems are listed in Additional file 4: Table S2. All antibodies for IHC were used in combination with the ultraView Universal DAB Detection Kit (VENTANA). Stainings were performed on the VENTANA BenchMark XT automated slide stainer using the “Cell Conditioning 1, Standard” setting for antigen retrieval and an incubation time of 16 min at 37 °C (estrogen and progesterone receptor) and 32 min (HER2) at 37 °C, respectively. HER2 SISH was also performed on the VENTANA BenchMark XT automated slide stainer with ISH-Protease 3 digestion for 8 min, followed by hybridization for 6 h and silver staining for 4 min. All slides were subsequently counterstained using the “Hematoxylin II” setting for 8 min and the “Bluing reagent” option for 4 min.

The results from central pathological investigation were used for further analyses.

### DNA extraction

We identified representative tumor areas using light microscopy of hematoxylin and eosin stained sections. If necessary, macrodissection was performed to reach a tumor cell content of at least 70%. Semi-automated DNA extraction was performed on the Maxwell RSC Instrument using the Maxwell RSC FFPE Plus DNA Purification Kit (Custom, AX4920; Promega), according to the manufacturer’s instructions. Extracted total DNA quantities were measured using the Qubit™ HS DNA Assay (Thermo Fisher Scientific).

### DNA methylation analysis

We used the Illumina Infinium HD FFPE DNA Restore Kit for DNA restoration of FFPE samples. Following restoration, the EpiTect Bisulfite Kit (Qiagen) was used for bisulfite conversion. DNA methylation analysis was performed using the Illumina Infinium MethylationEPIC BeadChip, according to protocols supplied by the manufacturer.

### Statistical analysis

Statistical analysis was performed using RStudio version 1.1.463 based on the statistical language R version 3.5.1. DNA methylation data were processed using the minfi package. The *combineArrays* function was used to merge data from the 450 K and 850 K array generation into a virtual 450 K dataset. The *pfilter* function of the wateRmelon package with the parameter perc = 5 was used to filter samples and probes with low quality [21]. Samples were preprocessed using the normal-exponential out-of-band (Noob) normalization. Furthermore, we used the *pwod* function of the wateRmelon package for probe outlier detection. Testing for differentially methylation positions (DMP) and differentially methylation regions (DMR) was performed using the *DMPfinder* function of the minfi package and the *bumphunter* function of the bumphunter package, respectively [22]. For both tests, hits with a false-discovery rate (FDR) below 0.05 and a median beta fold-change of at least 0.4 were considered to be significant. For the bumphunter function, we performed 1000 permutations.

T-distributed stochastic neighbor embedding (t-SNE) plots were generated based on the 5000 most variant CpG sites and using the *Rtsne* function of the Rtsne package with 2000 iterations while the perplexity was set to 25 [23]. Heatmaps were generated using the ComplexHeatmap package with the “ward.D2” method and “euclidean” distance measuring [24]. Tumor purity estimations for TCGA samples were derived from previously published datasets [25].

The means of Ki67 proliferation rates were tested for significance using the Wilcoxon–Mann–Whitney-Test. Pathological complete response (pCR) rates were compared using the Fisher’s test. Overall survival (OS) was visualized using Kaplan–Meier curves and tested for significant differences using pairwise logrank test.

## Supplementary Information

**Additional file 1. Fig. S1**: t-distributed stochastic neighbor embedding (t-SNE) plots showing the estimated tumor purity of The Cancer Genome Atlas samples based on manual estimation using light microscopy (a), RNAseq data (ESTIMATE; b), copy number variations (ABSOLUTE; c), DNA methylation (LUMP; d) and a combined score (CPE; e).

**Additional file 2. Fig. S2**: Flowchart showing the composition of the study cohort with samples from the publicly available “The Cancer Genome Atlas” (TCGA) dataset as well as specimens from the clinical GeparSixto and GeparSepto trial. Abbreviations: BRCA = breast cancer; ER = estrogen receptor; PR = progesterone receptor; TNBC = triple negative breast cancer; t-SNE = t-distributed stochastic neighbor embedding.

**Additional file 3. Table S1**: Sample annotation list for samples from the TCGA dataset.

**Additional file 4. Table S2**: Table displaying the antibodies used for immunohistochemistry and the corresponding scoring systems.

**Additional file 5. Table S3**: Results from DMR analysis comparing LowHR HRpos-like and TNBC samples

**Additional file 6. Table S4**: Results from DMR analysis comparing LowHR TNBC-like and TNBC samples

**Additional file 7. Table S5**: Results from DMR analysis comparing LowHR HRpos-like and LowHR TNBC-like samples

**Additional file 8. Table S6**: Correlation of clinicopathological metadata with DNA methylation groups

## Data Availability

Raw DNA methylation data of the samples analyzed in this paper have been deposited in GEO (GSE163521). To review GEO accession GSE163521: Go to https://www.ncbi.nlm.nih.gov/geo/query/acc.cgi?acc=GSE163521. Enter token kvinqqgylnwdhyb into the box.
