# Supplementary figures and images for: DNA methylation profiling identifies two distinct subgroups in breast cancers with low hormone receptor expression, mainly associated with HER2 amplification status
Source: Clin Epigenetics. 2021 Oct 3;13:184. doi: 10.1186/s13148-021-01176-5 (PMC8489064; doi:10.1186/s13148-021-01176-5)

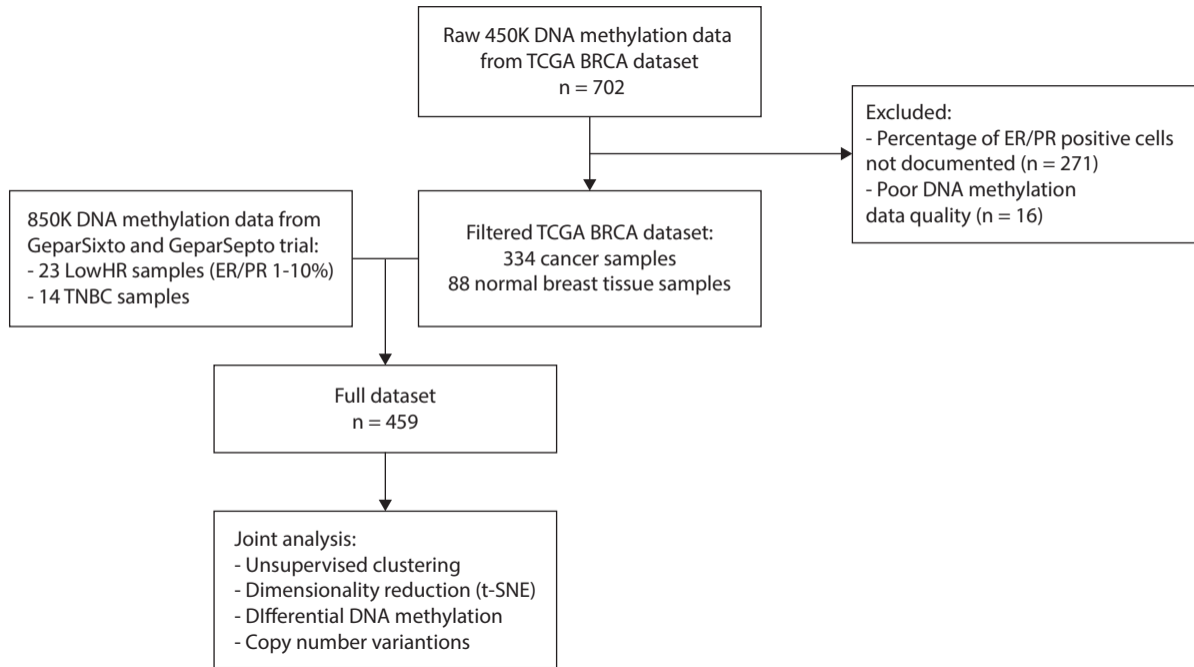

Supplement: Supplementary file 1 — Additional file 1. Fig. S1: t-distributed stochastic neighbor embedding (t-SNE) plots showing the estimated tumor purity of The Cancer Genome Atlas samples based on manual estimation using light microscopy (a), RNAseq data (ESTIMATE; b), copy number variations (ABSOLUTE; c), DNA methylation (LUMP; d) and a combined score (CPE; e). [file 13148_2021_1176_MOESM1_ESM.pdf]

**a**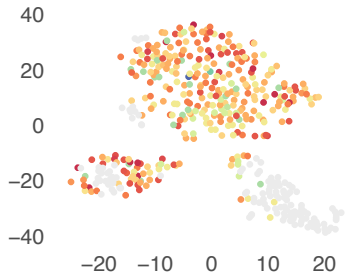**b**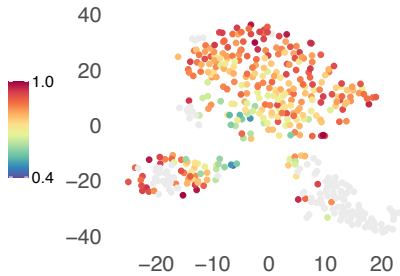**c**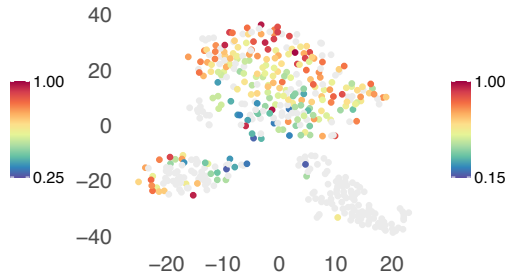**d**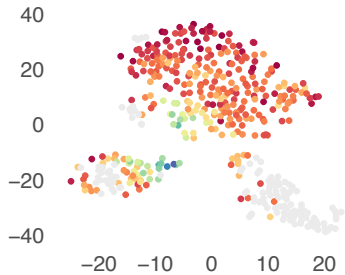**e**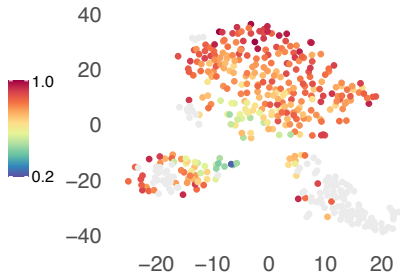

Supplement: Supplementary file 2 — Additional file 2. Fig. S2: Flowchart showing the composition of the study cohort with samples from the publicly available “The Cancer Genome Atlas” (TCGA) dataset as well as specimens from the clinical GeparSixto and GeparSepto trial. Abbreviations: BRCA = breast cancer; ER = estrogen receptor; PR = progesterone receptor; TNBC = triple negative breast cancer; t-SNE = t-distributed stochastic neighbor embedding. [file 13148_2021_1176_MOESM2_ESM.pdf]
