# Supplementary material for: DNA methylation profiling identifies two distinct subgroups in breast cancers with low hormone receptor expression, mainly associated with HER2 amplification status
Source: Clin Epigenetics. 2021 Oct 3;13:184. doi: 10.1186/s13148-021-01176-5 (PMC8489064; doi:10.1186/s13148-021-01176-5)
Supplement: Supplementary file 4 — Additional file 4. Table S2: Table displaying the antibodies used for immunohistochemistry and the corresponding scoring systems. [file 13148_2021_1176_MOESM4_ESM.docx]

| **Antigene** | **Manufacturer** | **Clone** | **Scoring** |
| --- | --- | --- | --- |
| Estrogen receptor | Ventana, USA | SP1 | Proportion of cells with nuclear staining of any intensity |
| HER2 immunohistochemistry | Ventana, USA | 4B5 | According to the ASCO CAP guidelines [5] |
| Ki67 | Dako, USA | Mib-1 | Proportion of cells with nuclear staining of any intensity |
| Progesterone receptor | Dako, USA | PgR636 | Proportion of cells with nuclear staining of any intensity |
| HER2 silver in-situ hybridization | Ventana, USA | - | According to the ASCO CAP guidelines [5] |

**Supplementary Table 2:** Table displaying the antibodies used for immunohistochemistry and the corresponding scoring systems.
